# Hair Microelement Profile as a Prognostic Tool in Parkinson’s Disease

**DOI:** 10.3390/toxics4040027

**Published:** 2016-11-16

**Authors:** Ferraro Stefano, Nasuti Cinzia, Piangerelli Marco, Guidi Marco, Giovannetti Rita, Ferri Augusto, Gabbianelli Rosita

**Affiliations:** 1 Chemistry Unit, School of Science and Technology, University of Camerino, Via S. Agostino 1, Camerino 62032, Italy; stefano.ferraro@unicam.it (F.S.); rita.giovannetti@unicam.it (G.R.); 2 Pharmacology Unit, School of Pharmacy via Gentile III da Varano, Camerino 62032, MC, Italy; cinzia.nasuti@unicam.it; 3 Computer Science Division, School of Science and Technology, University of Camerino, Via del Bastione 1, Camerino 62032, Italy; marco.piangerelli@unicam.it; 4 Neurology Unit, AOORMN, San Salvatore Hospital, Pesaro 61121, Italy; ma.guidi@ospedalimarchenord.it; 5 Well Dynamics Applications Srl, Reggio Emilia 42123, Italy; augusto.ferri@studiotricologico.eu; 6 Biochemistry and Molecular Biology Unit, School of Pharmacy Via gentile III da Varano, Camerino 62032, MC, Italy

**Keywords:** hair, microelements, biomarkers, Parkinson’s disease

## Abstract

Changes in the homeostasis of metals and microelements have been demonstrated in Parkinson’s disease, whose etiology includes both a genetic and environmental basis. We studied the difference of microelements in the hair of Parkinson’s disease subjects (*n* = 46) compared with healthy controls (*n* = 24). Hair was chosen as a representative matrix to measure microelements, since it is a vehicle of substance excretion from the human body and it allows for long-term evaluation of metal exposure. An inductively coupled plasma mass spectrometry (ICP-MS) analysis of hair collected from 24 Parkinson’s patients compared with their healthy relatives used as controls shows a significant decrease in Ca (*U = 166*, *p* = 0.012),), Mg (*U = 187*, *p* = 0.037), and Sr (*U = 183*, *p* = 0.030). Cd and Ca/Mg were decreased, and Cu was increased, in patients with respect to their healthy related controls at the limit of significance (*p* = 0.0501). Principal Component Analysis (PCA) of these microelements in hair shows a clustering into two groups according to gender, disease severity according to the Hoehn–Yahr scale, and pharmacological therapy. This pilot study represents a starting point for future investigations where a larger group of subjects will be involved to define other microelements useful when screening for early biomarkers of Parkinson’s disease.

## 1. Introduction

Parkinson’s disease (PD) is characterized in an incidence of 5%–10% from genetic alterations; hence, the etiology of this neurodegeneration can be associated to an interplay between genes and environmental factors that can modulate gene expression and protein metabolism [1]. Furthermore, a correlation between idiopathic PD and early onset of the disease has been observed not only in genetic forms but also in exposed occupational subjects with no family history of the disease among their first-degree relatives, highlighting the key role of environmental factors in the development of Parkinson’s disease [2]. At the same time, epidemiological investigations on humans and studies on animal models of Parkinson’s-like diseases have shown that metals, neurotoxicants, and pesticides play a key role in the onset of PD [2,3,4,5,6,7,8,9,10,11,12,13]. Metals can accumulate in microscopic proteinopathies leading simultaneously to their decrease in cellular microenvironments, where they play a key role in biological processes [14]. Iron accumulation in specific basal ganglia has been reported in Parkinsonism together with a concomitant low plasma level of the metal, since iron retention in the brain could be associated with the failure of iron passage from brain to plasma [14]. Oxidative stress and the increase in alpha-synuclein are linked to iron, since this metal is required in the Fenton reaction and as an enhancer of protein synthesis, respectively [14]. Like iron, other important biological metals such as Cu, Zn, and Mn are imbalanced in PD [1,2,4,5,6,13,14].

A key point in the evaluation of microelement changes is the matrix to be used to detect “chronic” imbalances in the body. Plasma and urine represent an appropriate matrix for the measurement of daily microelements present in the human body because of homeostasis processes, whereas 2 cm long hair reflects what has been in the blood stream for the last 30 days [15]. Furthermore, the level of microelements in hair is up to 10-fold higher than that found in blood or urine [16]. This is mainly due to the presence of cystine and metal cations that form bonds with the sulfur of the matrix hair keratin and to the accumulation of microelements over a longer time period [17]. For this reason, hair represents an attractive choice for occupational and environmental surveys. Moreover, hair has the following advantages: (1) it is a stable matrix; (2) its collection, transportation, and storage is far simpler; (3) it is a biological material that can be collected in a much less invasive manner than blood samples; (4) hair can be segmented in order to determine repeated measurements over time. The Environmental Protection Agency (EPA) has accepted the use of human hair as a matrix for environmental monitoring [18].

Recently, we showed that in an animal model of Parkinson’s-like disease, there was a significant increase in As, Mg, S, and Zn in the hair of 12-month-old rats compared with 6-month-old ones, corresponding to an approximate relative human lifespan of 30 and 18 years old, respectively [19]. In addition, in the same animal model of PD, K, Si, and the Cu/Zn ratio were decreased [19]. Furthermore, several data emphasize the interest in research based on the microelement profile in hair as a way to identify early biomarkers of several diseases, as reported with autism spectrum disorder and in an animal model of age-related diseases [20,21,22]. In PD, knowledge of biomarkers before clinical manifestations of the disease represents a major research goal since Parkinsonism symptoms appear once 50% of dopamine neurons have already died [23]. For this objective, the deterioration in the sense of smell has been suggested [24]; however, as reported in an animal model of Parkinson’s-like disease [19], hair analysis could be useful for the screening of microelement changes as early biomarkers in human hair because these microelements could be modified, demonstrated in an animal model of Parkinson’s disease before the death of 50% of dopaminergic neurons [19].

With the aim to provide new insights on biomarkers for Parkinson’s disease, we investigated the difference in microelements (Na, Mg, Al, Si, P, S, K, Ca, Cr, Mn, Fe, Ni, Cu, Zn, As, Se, Rb, Sr, Ag, Cd, Sn, Sb, Hg, Pb, and Ca/Mg) in the hair of PD subjects compared with healthy controls. For this purpose, we collected and analyzed via ICP-MS hair from volunteers with and without a diagnosis of PD. A restricted control subject and a PD subject were selected from within a single family, taking hair from the couple (e.g., a wife and a husband). Microelement clustering in the two family groups permitted the identification, following PCA, of which microelements in the hair was modified according to gender, the importance of the disease, pharmacological therapy, and the Hoehn–Yahr scale.

## 2. Materials and Methods

### 2.1. Recruitment of Participants

A total of 70 volunteers were recruited in this case-control study after informed consent was given by the participants.

Of these, 46 (30 males and 16 females) had a diagnosis of PD according to Movement Disorder Society (MDS) Clinical Diagnostic Criteria [25], and 24 (8 males and 16 females) were healthy relatives used as controls with no history of neurological disease or exposure to environmental metals. The exclusion criteria for PD patients were a history of repeated strokes with stepwise progression, a previous head injury, use of antipsychotic or dopamine depleting drugs, definite encephalitis, oculogyric crises, or both on no drug treatment, a negative response to large doses of levodopa, and other neurological features such as supranuclear gaze palsy, cerebellar signs, early severe autonomic involvement, Babinski signs, occupational exposure to metals, and communicating hydrocephalus on neuroimaging.

The history of each patient was collected in order to obtain information on the onset of clinical manifestations, disease duration, and disease severity using the Hoehn–Yahr scale [26], as well as the type of pharmacological treatment.

In the PD group, the mean age was 72.33 years (SEM = 1.25 years); in the healthy related controls, the mean age was 68.25 years (SEM = 1.83 years).

In order to compare data on PD subjects with healthy controls from the same family, 24 PD patients (17 males and 7 females) were selected from the 46 subjects recruited. The control subject from the same family was the wife or the husband of the PD patient. Their mean age was 71.29 (SEM = 1.62).

Participant recruitment was carried out at the Neurology Unit in San Salvatore Hospital (Pesaro, Italy) after informed consent.

### 2.2. Hair Collection

Hair samples were collected by the same operator throughout the project at the Neurology Unit in San Salvatore Hospital (Pesaro, Italy) and Cuore Salus (Fabriano, Italy), and a number was assigned to each sample in order to operate under blind conditions for the hair analysis.

Hair samples were washed 24–48 h prior to collection with a neutral shampoo. Ten hairs from the bulb with a maximum length of 2 cm were collected at the frontoparietal, occipital, and retroauricular areas in the left and right part of the head, obtaining 60 hair samples per person.

### 2.3. Hair Analysis

Twenty-five macro- and microelements, including heavy metals (Na, Mg, Al, Si, P, S, K, Ca, Cr, Mn, Fe, Ni, Cu, Zn, As, Se, Rb, Sr, Ag, Cd, Sn, Sb, Hg, Pb, and Ca/Mg), were analyzed via the ICP-MS technique [27]. This validated method is widely used for hair analysis [28,29]. Hair samples were cut into small pieces using a clean ceramic knife. About 60 ± 7 mg were transferred into a polyethylene-labeled weighing pan, and the exact weight was recorded. To each sample, 5 mL of reagent-grade nitric acid (HNO_3_) were added as an oxidant solution to the Teflon vessel (Berghof Speedwave 4), and the samples were then incubated for 10 min prior to the onset of mineralization [29,30]. After the mineralization process, the samples were cooled down to ambient temperature, and the solution was transferred to 10 mL polyethylene test tubes and filled up with reagent grade water type 1. One milliliter of the solution was transferred to a test tube and diluted 10 times with reagent grade water type 1 in order to decrease the acid concentration. The solution was analyzed for the amounts of mineral elements and trace metals via ICP-MS. Sample results were quantified by multiple calibration curves for all elements, and the results were checked by comparison with Certified Reference Material Hairs (ERM DB001, GBW 07601) treated in the same mode.

### 2.4. Statistical Analysis

Statistical analysis was carried out using the program Statistica 8.0 (StatSoft Italy Srl, Vigonza, PD, Italy, 2007). Since data of all microelements were not normally distributed, Mann–Whitney was employed for analysis. Differences were considered significant at a *p*-value of 0.05.

### 2.5. Principal Component Analysis and Hierarchical Cluster Analysis

Besides statistical data analysis, data were analyzed using two of the golden standard techniques in this field: Principal Component Analysis (PCA) and Hierarchical Clustering (HC). PCA is an unsupervised statistical procedure used both for reducing the dimensionality of multivariate data and for obtaining information on the possible clusters in the data which could be hidden by their high dimensionality. Technically, PCA transforms the data projecting them into a new space, which is built using a new basis in such a way where the maximal variance is exposed. The new orthonormal coordinate system of data is made up by the so-called Principal Components (PCs): they are mutually orthogonal and allow for a rationalization of the maximal variance.

Clustering is a methodology for classifying a finite set of objects or data. Data are represented using a proximity matrix where, by using a metric, the “proximities” among the data are recorded. The proximity matrix is the input of any clustering algorithm. In this paper, we used the agglomerative hierarchical clustering approach. This method can be either bottom-up or top-down: in the first case, it consists of building nested clusters (hierarchical clusters), where clusters at level i are the fusion of (agglomerative clustering) clusters at level i – 1; in the second case, the clustering at level i is given by the splitting up (division clustering) of a bigger cluster at level i + 1.

In this paper, we performed the agglomerative clustering using the R package “stats.” In detail, the agglomerative clustering operates according to the following steps:

1. At the beginning, we have a collection of N atomic cluster, and each cluster contains one data point.
2. According to a linkage method, the closest clusters are found.
3. The closest clusters are melted into new clusters.
4. The melted clusters are removed from the collection of clusters.

Steps 2, 3, and 4 are repeated until only one cluster remains; at the end, the procedure produces a dendrogram: a hierarchical tree of clusters.

A fundamental point in this procedure is to define a linkage method, a method to measure the distance between two clusters.

Both PCA and HC were performed using the free software R (Version 3.2.2, R CoreTeam, Vienna, Austria) under GNU General Public License, www.r-project.org.

## 3. Results

### 3.1. Hair Analysis

ICP-MS analysis on PD patients (*n* = 46) shows that Ca, Sr, and Cd decrease significantly with respect to the healthy control group (*n* = 24) (Table 1). If we select only PD patients (*n* = 24) from the same family of healthy controls (*n* = 24), their hair, compared with that of their relatives as controls, shows a significant decrease in Ca (*U = 166*, *p* = 0.012), Mg (*U = 187*, *p* = 0.037), and Sr (*U = 183*, *p* = 0.030). Cd was decreased in PD patients with respect to healthy related controls at the limit of significance (*p* = 0.0501) (Table 1).

### 3.2. PCA

PCA was performed taking into account the data of six metals/microelements (Ca, Cd, Sr, Mg, Ca/Mg, and Cu) obtained from only 48 samples, i.e., 24 controls (C) and 24 PD patients.

**Figure 1:**
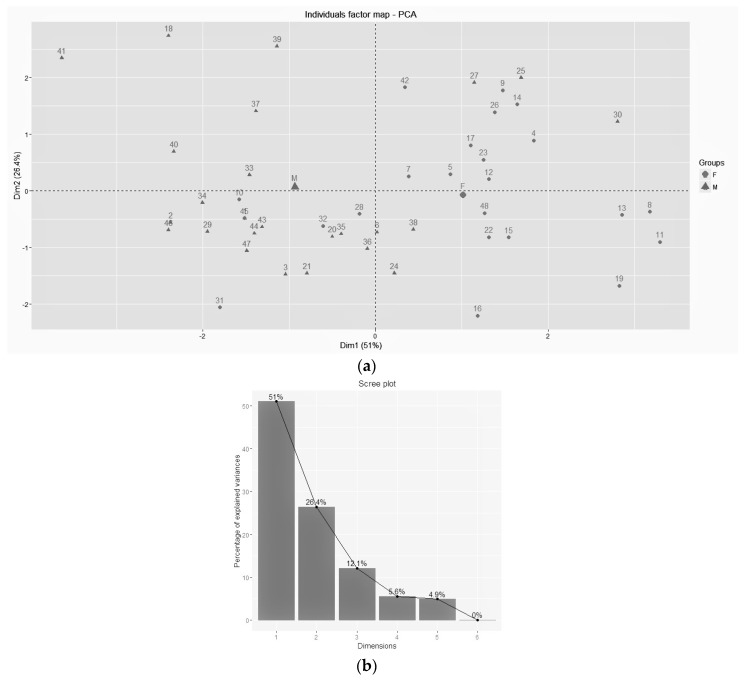
Principal Component Analysis (PCA) (**a**) of subjects clustered according to gender (F = ⚫; M = ▲) and screen plot (**b**).

From the control group, we took into account only healthy people paired with PD patients (husband and wife) in order to reduce the variability between subjects in the hope of removing some of the environmental differences. For PCA, the six microelements that were statistically significant or near statistical significance were used. The results of the PCA are shown in Figure 1. According to the screen plot 1B, only the first two PCAs were used because they are able to explain 77% of total variance. In Figure 1A, we can see the effect of PCA on our data set: patients are clustered according to the gender type into two well-defined groups (left: male; right: female).

### 3.3. Hierarchical Clustering Analysis

**Figure 2:**
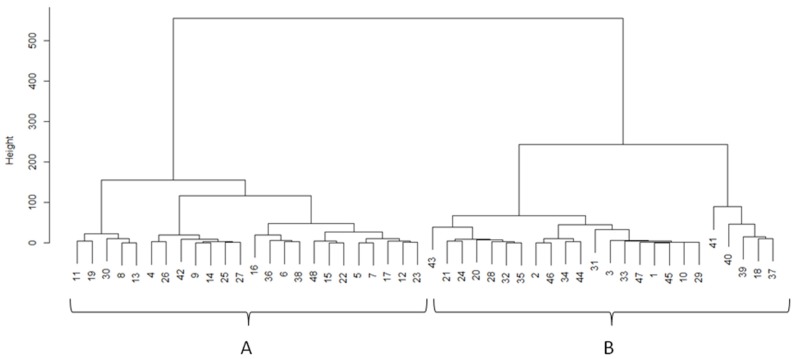
Cluster dendrogram obtained from PD (*n* = 24) and healthy subjects from the same family (*n* = 24). Cluster A includes healthy controls and mainly females with low grade of PD, while Cluster B includes mainly male PD patients with a severe grade of PD and who had a longer and stronger medication.

The same data (Ca, Cd, Sr, Mg, Ca/Mg, and Cu) processed with PCA were analyzed by Ward’s method equipped with the squared Euclidean metric [31]. This is the standard way to use the *hclust* command in *R*. In fact, in the classical formulation of Ward’s method, given two clusters, $A={\left\{x_{i}\right\}}_{i=1}^{n_{A}}$ and $B={\left\{x_{j}\right\}}_{j=1}^{n_{B}}$, the distance between them is given by

(1) $$d_{W}=\frac{n_{A}n_{B}}{n_{A}+n_{B}}{\left\|\rho -\mu \right\|}_{2}^{2}$$

where ρ and μ are the centroids of the Clusters *A* and *B* (Figure 2).

## 4. Discussion

Dysregulation of metal and microelement homeostasis has been reported in several neurodegenerative diseases [19,20,21,22,32]. In particular, deficits in Ca and Mg have been associated with neuronal disorders; Ca plays a key role in signal transduction, while Mg is a key cofactor of many enzymes influencing neurotransmission through regulation of their mediators [33]. Furthermore, a deficit in Mg has been associated with depression and neuromuscular transition deficits [33]. Rats fed low levels of Mg in early life have shown decreases in dopaminergic neurons together with activation of microglia in substantia nigra pars compacta [33]. Low Mg dietary intake has been associated with deficits in the olfactory function typically present in PD patients, and Mg supplementation has had positive effects against PD in the Japanese population [33]. On the other hand, Mg and Ca deficiency has been associated with the first signs of neurological and neuromuscular disturbances in neurodegeneration, together with the accumulation of toxic metals like Cd, which is linked to oxidative stress and superoxide dismutase inhibition [32,33]. In addition, Cd, Pb, and As have also been reported to induce damage at the blood–brain barrier (BBB) following early life exposure [34].

In neurodegenerative disorders, abnormal metal–protein interactions could lead to damage of the BBB or energy imbalances in the brain because the metal regulatory transport system depends on ATPase activity [35,36,37,38]. This point indicates that metal dyshomeostasis may occur even in subjects that are not exposed to toxic environments [38]. In our study, a significantly lower content of Ca and Mg, together with a lower level of Cd at the limit of significance, were detected in the hair of PD patients compared with healthy related controls, the wife or husband living in the same family. The comparison with the healthy spouse permits the comparison of subjects with a similar life style and age. An interesting outcome obtained in this study through the Hierarchical Clustering Analysis was that subjects with mild Parkinsonism were grouped together and were distinct from the PD group with more severe symptoms. Cluster A was comprised mainly of female PD patients with mild symptoms and low dosage of dopaminergic treatment, while Cluster B included mainly male PD patients with more serious symptoms and longer pharmacological treatment. In addition, the subjects clustered in Cluster A have a level of Ca, Mg, Sr, and Cd in their hair similar to the healthy controls. This therefore suggests that Ca, Mg, Sr, and Cd represent potential biomarkers that distinguish the severity of the disease.

## 5. Conclusions

In conclusion, this study shows that metals and microelements are imbalanced in hair from PD patients compared with controls, and that different genders and levels of disease severity can discriminate PD subjects that cluster in different groups. Further studies are required to generate data on larger numbers of patients in order to develop a model, based on specific algorithms, that is useful for calculating the cut-off value of selected metals and microelements, differentiating subjects developing PD from healthy ones. The next step will be to elaborate a predictive model to classify data that could be useful for clustering groups according to disease severity. This classification can bring new insights on microelement changes and could lead to new therapeutic approaches to contrast PD progression.

## Figures and Tables

**Table 1:** Metals and microelements in the hair of Parkinson’s patients and control subjects.

Metals and Microelements (ppm)	Control (*n* = 24) ^#^ Mean ± SEM	PD (*n* = 24) ^#^ Mean ± SEM	PD (*n* = 46) Mean ± SEM
Na	727.018 ± 125.760	620.395 ± 92.464	628.611 ± 92.464
Mg	207.201 ± 30.075	153.617 * ± 24.717	174.294 ± 27.013
Al	111.452 ± 22.754	262.138 ± 64.209	283.230 ± 66.506
Si	303.830 ± 62.176	308.630 ±32.806	335.799 ± 41.869
P	367.064 ± 42.159	538.565 ± 59.665	503.966 ± 53.817
S	42372.277 ± 1205.134	41186.553 ± 954.733	40144.768 ± 1037.435
K	855.179 ± 142.877	1008.183 ± 127.685	1014.116 ± 125.460
Ca	2309.983 ± 350.837	1361.330 * ± 237.619	1674.490 * ± 266.350
Cr	2.633 ± 1.745	0.937 ± 0.113	1.341 ± 0.211
Mn	0.255± 0.032	0.195± 0.032	1.775 ± 0.591
Fe	15.174 ± 2.119	11.643 ± 1.156	24.812 ± 3.064
Ni	2.018 ± 0.388	3.162 ± 0.890	2.846 ± 0.713
Cu	25.276± 9.854	16.900± 3.011	16.467 ± 2.925
Zn	126.588± 9.575	103.297± 9.310	177.398 ± 12.844
As	0.024 ± 0.004	0.027 ± 0.002	0.024 ± 0.002
Se	0.522 ± 0.093	0.462 ± 0.022	0.428 ± 0.023
Rb	0.932 ± 0.162	1.495 ± 0.217	1.350 ± 0.197
Sr	14.034 ± 2.700	7.297 * ± 1.289	8.367 * ± 1.844
Ag	0.102 ± 0.025	0.093 ± 0.016	0.097 ± 0.016
Cd	0.024 ± 0.006	0.009 ^§^± 0.001	0.009 * ± 0.001
Sn	0.670 ± 0.188	0.446 ± 0.146	0.417 ± 0.112
Sb	0.075 ± 0.015	0.103 ± 0.045	0.196 ± 0.113
Hg	9.665 ± 3.113	6.552 ± 1.026	4.588 ± 0.844
Pb	1.038 ± 0.241	0.735 ± 0.083	0.627 ± 0.069
Ca/Mg	12.210 ± 2.006	10.721 ± 1.503	11.834 ± 1.823

^#^ Selected from the same family (wife and husband). * *p* < 0.05; ^§^ *p* = 0.05.
